# In Vitro Propagation, Phytochemical Analysis, and Evaluation of Free Radical Scavenging Property of *Scrophularia kakudensis* Franch Tissue Extracts

**DOI:** 10.1155/2015/480564

**Published:** 2015-11-16

**Authors:** Abinaya Manivannan, Prabhakaran Soundararajan, Yoo Gyeong Park, Byoung Ryong Jeong

**Affiliations:** ^1^Division of Applied Life Science (BK21 Plus), Gyeongsang National University, Jinju 660-701, Republic of Korea; ^2^Institute of Agriculture and Life Science, Gyeongsang National University, Jinju 660-701, Republic of Korea; ^3^Research Institute of Life Science, Gyeongsang National University, Jinju 660-701, Republic of Korea

## Abstract

The current study deals with in vitro propagation, antioxidant property estimation, and assessment of acacetin content in *Scrophularia kakudensis* Franch. Adventitious shoot induction was achieved from the nodal explant with the highest number of adventitious shoots per explant (17.4) on Murashige and Skoog's (MS) medium fortified with 2.0 mg·L^−1^ 6-benzyladenine (BA) and 0.5 mg L^−1^ indole-3-acetic acid (IAA). Maximum number of roots per plant (16.5) was noted in half strength MS medium supplemented with 0.5 mg·L^−1^ IAA. The regenerated plants displayed successful survival ratio (95%) in the greenhouse. The highest content of acacetin, a pharmaceutically important flavonoid, was observed in the shoot extracts (in vitro: 32.83 *µ*g·g^−1^ FW; in vivo: 30.05 *µ*g·g^−1^ FW) followed by root extracts. Total phenol and flavonoid contents along with free radical scavenging assays revealed the occurrence of larger amount of antioxidants in shoot extract in comparison with callus and root extracts of *S. kakudensis*. Thus, the outcome of the present study can be highly beneficial for the germplasm conservation and commercial cultivation of *S. kakudensis* for therapeutic purposes.

## 1. Introduction

The genus* Scrophularia* (Scrophulariaceae) consists of 300 species of medicinal plants with notable pharmaceutical importance against several diseases such as inflammation, fever, and gastrointestinal problems [1].* Scrophularia kakudensis* Franch is a potential medicinal plant species distributed around the mountains of Korea, Japan, and China with pharmaceutically important secondary metabolites such as acacetin [2] and scrophulasaponins [3]. The morphological characteristics of* S. kakudensis* include quadrangular, white pilose stem, ovate to narrowly ovate leaf blade, glandular hairy peduncles, and broadly ovoid capsules [4]. Besides its medicinal importance,* S. kakudensis* is still unexplored due to inadequate healthy plant materials, narrow environmental adaptation, and risk of extinction. Domestication of* Scrophularia* sp. including* S. kakudensis* Franch is hindered by seed dormancy [5]. Nevertheless, the above-mentioned difficulties can be resolved by plant tissue culture approaches. In recent years, plant tissue culture has developed into a powerful tool for mass propagation, conservation, cryopreservation, and genetic manifestations of medicinal plants [6, 7]. Thus, the first objective of the current work is to devise an efficient mass propagation protocol for* S. kakudensis*.

As mentioned above the* S. kakudensis* plants are known for the presence of important secondary metabolite acacetin. Acacetin is a 5, 7-dihydroxy-4′-methoxyflavone, reported with several therapeutic effects such as anticancerous, antidiabetic, antipyretic, antiperoxidative, anti-inflammatory, antiplasmodial, and antiproliferative activities by inducing apoptosis and blocking the progression of cell cycles [8–13]. Moreover, our recent in silico analysis revealed the effective binding of acacetin towards the aldose reductase enzyme, a vital drug target in diabetic and cancer treatment [14]. Though the presence of acacetin has been demonstrated by Kim et al. [2] in* S. kakudensis*, for the first time in the current work the content of acacetin in shoot and root extracts of in vitro plantlets of* S. kakudensis* has been determined and compared with the in vivo extracts. In addition, the antioxidant potentials of in vitro tissues were also examined to gain insight into the free radical scavenging nature of* S. kakudensis* extracts. Antioxidants are compounds that inhibit the substrate oxidation possibly by acting as the free radical scavenger. Broadly, most of the medicinally important bioactive secondary metabolites are phenols and flavonoids. These therapeutic metabolites prevent several life threatening degenerative diseases such as cancer, cardiovascular, and neurological disorders caused by oxidative stress [15]. The oxidative stress leads to the excess production of highly reactive oxygen species (ROS) that are extremely harmful to cells. Among ROS, hydrogen peroxide, superoxide, nitric oxide, and hydroxyl ions are the most notable radicals detrimental to the cells [16]. Eventually, the increase in the ROS level can lead to cell death by oxidation of the biomacromolecules such as protein, DNA, and unsaturated fatty acids [17]. Therefore, it is necessary to determine the plant tissue containing large amount of antioxidants for the characterization and identification of novel lead molecules for drug discovery purposes. Overall, the current study has established an efficient in vitro propagation procedure followed by the characterization of antioxidant properties of in vitro plants in comparison with greenhouse grown in vivo plants.

## 2. Materials and Methods

### 2.1. Plant Material and Culture Condition

Seeds of* S. kakudensis* obtained from the Wild Plant Seed Bank, Daejeon, Korea, were decontaminated with 80% (v/v) ethanol for 30 seconds followed by 1.0% (v/v) sodium hypochlorite (NaClO) for 4 min. Excess NaClO was washed off in distilled water (dis. H_2_O) for 5–7 times and then the seeds were blot dried. After sterilization, seeds were sown on the half strength Murashige and Skoog (MS) [18] basal medium with 3% (w/v) sucrose and 0.8% (w/v) agar. The pH of the medium was adjusted to 5.75 using 0.1 N NaOH or 0.1 N HCl and autoclaved at 121°C for 15 min. After 5 weeks, nodal segments obtained from a single seedling were subcultured in the plant growth regulator- (PGR-) free MS medium until enough explants were acquired for micropropagation. All the cultures were maintained at 25°C and 80% relative humidity (RH) under a 16 h photoperiod with 50 *μ*mol m^−2^ s^−1^ photosynthetic photon flux density (PPFD) provided by cool white fluorescent light (40 W tubes, Philips, Netherlands).

### 2.2. Adventitious Shoot Induction and Proliferation

Nodal explants from four-week-old shoots were inoculated on the MS medium supplemented with various concentrations (0.0, 0.5, 1.0, 2.0, or 3.0 mg L^−1^) of 6-benzyladenine (BA), kinetin (Kn), and thidiazuron (TDZ) alone or in combination with 0.5 or 1.0 mg L^−1^ indole acetic acid (IAA) for adventitious shoot induction. Thidiazuron was filter-sterilized and added to the autoclaved medium, whereas other plant growth regulators (PGRs) were added to the basal medium prior to pH adjustment and sterilization. After five weeks of inoculation, shoot bud induction percentage and average number of shoots induced per explant were recorded. In each treatment, 30 explants were used and the experiment was repeated thrice for reproducibility.

### 2.3. Shoot Elongation, In Vitro Rooting, and Acclimatization

The clump of microshoots were separated and transferred to MS medium devoid of PGRs for shoot elongation. After three weeks, the elongated shoots (2–4 cm) were transferred to half strength or full strength MS medium with or without 0.5 mg·L^−1^ or 1.0 mg·L^−1^ of IAA, indole-3-butyric acid (IBA), and *α*-naphthalene acetic acid (NAA). After three weeks, root induction percentage, number of roots per plant, and length of the longest root were measured. Plantlets with well-developed roots were transplanted into 10 cm pots containing a greenhouse growing medium (Tosilee Medium, Shian Precision Co., Jinju, Korea) and grown in a glasshouse at Gyeongsang National University, Jinju, Korea, under a normal day light condition with night/day set temperatures of 27/19°C and 60–70% RH. The survival ratios of the micropropagated plants were recorded after five weeks of acclimatization.

### 2.4. Estimation of Phytochemicals in Tissue Extracts

#### 2.4.1. Preparation of Plant Extracts

For phytochemical analysis and antioxidant capacity assessment, 4-week-old in vitro plants cultured in MS medium without PGRs, callus (4 weeks old) induced by 3.0 mg·L^−1^ BA, and 4-week-old greenhouse grown in vivo plants (separated into shoot and root) were fractioned with methanol according to Manivannan et al. [19]. Briefly, the plant samples (0.5 g) were lyophilized and extracted with 5 mL of 80% (v/v) methanol overnight under 150 rpm in a rotating shaker. The resulting homogenates were centrifuged at 12,000 rpm for 10 min and the supernatant was employed for the in vitro assays.

#### 2.4.2. Total Phenol and Flavonoid Estimation

Total phenol content of the extracts was estimated by Folin-Ciocalteu principle according to the procedure of Manivannan et al. [19]. Aliquot of the extracts (0.1 mL) made up to 1 mL with distilled water was mixed with 0.5 mL of Folin-Ciocalteu reagent (1 : 1 with water) and 2.5 mL of sodium carbonate solution (7.5%). The reaction mixture was vortexed vigorously and incubated in the dark for 40 min. After incubation the absorbance was recorded at 725 nm and the total phenol content was expressed as gallic acid equivalents (GAE). The total flavonoid composition was determined based on the aluminum chloride calorimetric method as mentioned by Ordoñez et al. [20] with slight modifications. Samples (0.1 mL) were made up to 1 mL with 80% methanol and used for the analysis by adding 1 mL of 2% aluminum chloride solution. The absorbance of the reaction mixture was measured at 415 nm after 30 min incubation and the total flavonoids were calculated from the standard quercetin calibration curve.

#### 2.4.3. Quantification of Acacetin Using High Performance Liquid Chromatography (HPLC)

The plant extract preparation and estimation of acacetin were carried out according to the procedure outlined by Yang et al. [21]. Briefly, the samples (5 g) were lyophilized and refluxed in 50 mL methanol for 24 h in rotatory shaker at 150 rpm and concentrated under reduced pressure. The concentrated extracts were filtered in 0.45 *μ*M syringe filter prior to chromatographic analysis in a 1200 series HPLC instrument with diode array detector (DAD) (Waters, MA, USA). The mobile phase consisted of acetonitrile (100% solvent A) and 1.0% glacial acetic acid (solvent B). The chromatographic separation was performed with solvent proportion of 37 : 63 using a ODS HYPERSIL column (4.6 × 250 mm, 5 *μ*M) with 1.25 mL·min^−1^ flow rate with 10 *μ*L sample injection volume. The absorbance of the standards and samples was recorded at 326 nm. The quantity of acacetin content was elucidated from the standard calibration curve.

### 2.5. Antioxidant Properties of Tissue Extracts

#### 2.5.1. Superoxide (O_2_^−^) Radical Scavenging Assay

Superoxide scavenging activity of the extracts was determined based on the ability of the extracts to inhibit formazan production by bleaching the superoxide radicals generated by nitroblue tetrazolium salt with riboflavin and light [16]. The extracts (0.1 mL) were added to the reaction mixture (0.1 mg NBT, 12 mM EDTA, and 20 *μ*g riboflavin in 50 mM sodium phosphate buffer (pH 7.6)) and illuminated by light. After 90 seconds the absorbance was measured at 590 nm.

#### 2.5.2. Nitric Oxide (NO) Radical Scavenging Assay

In this assay the inhibition of NO production by the extracts was determined using sodium nitroprusside (SNP) mediated generation of nitric oxide [16]. The nitric oxide spontaneously produced by SNP reacts with the oxygen to form nitrite ions that can be measured by Griess reagent. The reaction was initiated by the addition of 10 mM SNP in phosphate buffered saline to the extracts (0.1 mL) and allowed to stand for 150 min at room temperature. After incubation, 0.5 mL of freshly prepared Griess reagent (2% phosphoric acid, 1% sulfanilamide, and 0.1% N-(1-naphthyl) ethylenediamine dihydrochloride) was added and the absorbance was determined at 546 nm.

#### 2.5.3. Hydrogen Peroxide (H_2_O_2_) Radical Scavenging Assay

For H_2_O_2_ scavenging assay, 0.6 mL of H_2_O_2_ (2 mM) was mixed with the extracts and incubated for 10 min. The absorbance was noted at 230 nm against a blank solution devoid of H_2_O_2_ according to the method described by Kumaran and Joel Karunakaran [16].

#### 2.5.4. 2,2-Diphenyl-1-picrylhydrazyl (DPPH) Radical Scavenging

The stable DPPH radical scavenging ability of the extracts was analyzed by mixing sample extracts (40 *μ*L) to 1,960 *μ*L of 0.1 mM methanolic solution of DPPH and allowed to stand for 25 min under dark conditions. The absorbance of the sample was measured at 517 nm according to Manivannan et al. [19].

For all the free radical scavenging assay ascorbic acid was employed as the standard. The radical scavenging % was calculated using the formula [(*A*_*c*_ − *A*_*s*_)/*A*_*c*_]*∗*100, where *A*_*c*_ is the absorbance value of the control (reaction mixture without extract) and *A*_*s*_ is the OD value of the extract or ascorbic acid.

#### 2.5.5. Hydroxyl Radicals Induced Plasmid DNA Strand Scission

The DNA protection ability of the extracts was determined by hydroxyl radicals mediated strand scission assay according to Abbas et al. [22]. Briefly, the pET 28 plasmid DNA (0.5 *μ*g) was mixed with FeSO_4_ (1 mM, 2 *μ*L), extract or ascorbic acid (2 *μ*L), H_2_O_2_ (20 mM, 4 *μ*L), and phosphate buffer (50 mM, pH 7.0, 2 *μ*L) and incubated in 37°C for 1 h. The extent of DNA damage protection was analyzed using agarose gel electrophoresis.

### 2.6. Statistical Analysis

All the experiments were set up in a completely randomized design with three replications per treatment and the assays were performed in triplicate to verify the reproducibility of the results. Significant differences among the treatments were determined by analysis of variance (ANOVA) followed by Duncan multiple range tests at a 5% probability level by using SAS computer package (SAS Institute Inc., NC, USA).

## 3. Results and Discussion

### 3.1. Adventitious Shoot Induction and Proliferation

**Figure 1:**
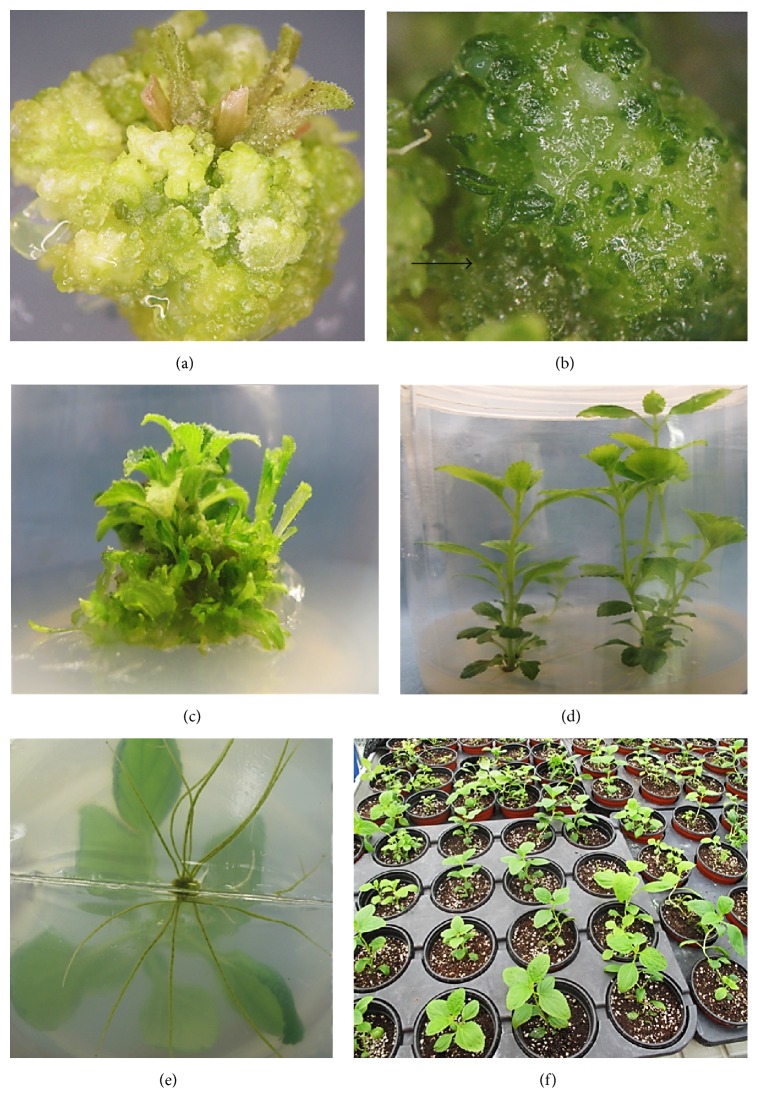
Adventitious shoot organogenesis in* S. kakudensis* Franch. (a) Clump of green colored protuberances appeared from the nodal explant. (b) Early stage of adventitious shoot induction with small leaves indicated by an arrow. (c) Adventitious shoots induced after three weeks. (d) Shoot elongation. (e) In vitro root induction from the shoot base. (f) Acclimatized plants in a greenhouse.

In the present investigation, adventitious shoot organogenesis was established from nodal explants derived from the axenic in vitro grown shoots by different concentrations and combinations of PGRs. Initially, the seeds were germinated in vitro and the seedlings were grown in PGR-free MS medium. After three subcultures in four-week interval, the nodal explants were excised and inoculated on the hormone-free MS medium (control), MS medium containing different concentrations, and combinations of PGRs for shoot multiplication. Explants cultured on the PGR-free MS medium produced only a single shoot per explant. Similarly, the explant cultured on shoot induction medium supplemented with three different cytokinins produced several adventitious shoots. Initially, the nodal explants cultured on the cytokinins-fortified medium were bloated within 10–12 days and the adventitious shoots appeared as small multiple protuberances on the cut surfaces (Figure 1(a)). After two weeks, caulogenesis was identified with small leaf-like structures observed from the outgrowths which subsequently developed into adventitious shoots (Figure 1(b)).

Among the cytokinins tested, the greatest frequency (100%) of shoot induction and number of shoots (12.4) were obtained on the medium supplemented with 2.0 mg L^−1^ BA (Table 1). However, increase in concentration of BA to 3.0 mg L^−1^ decreased the shoot induction frequency and led to the formation of dark green colored complex calli on the leaf edges. The exceptional explant response to BA can be due to its rapid metabolism and ability to activate other endogenous hormones inside the plant tissue [23]. Similarly, the positive role of BA in shoot induction was also noted on other species of* Scrophularia* such as* Scrophularia takesimensis* [24] and* Scrophularia yoshimurae* [1]. Moreover, 2.0 mg·L^−1^ Kn also induced the greatest shoot induction frequency (100%), but the number of shoots formed per explant (8.5) was lesser as compared to the BA treatment. Callus formation at the nodal base appeared on the TDZ-supplemented medium after 13 days. In addition, most of the adventitious shoots on the TDZ-supplemented medium were hyperhydric irrespective of its concentration. Hyperhydricity refers to the physiological malformation of the regenerated shoots that result in excessive hydration, low lignification, and poor regeneration. Appearances of hyperhydricity in shoots are generally characterized by several stress conditions during the process of tissue culture and also due to the usage of higher concentrations of cytokinins such as TDZ [25]. Generally, cytokinins used in combination with auxin enhance the adventitious shoot induction [26]. In the current study, addition of IAA (0.5 or 1.0 mg·L^−1^) along with BA (0.5–3.0 mg·L^−1^) has gradually enhanced the number of shoots induced per explant as compared to the medium containing BA alone (Table 2). The greatest number of shoots per explant (17.4) was obtained on the MS medium containing 2.0 mg·L^−1^ BA and 0.5 mg·L^−1^ IAA (Figure 1(c)). However, the adventitious shoot elongation was hindered in the shoot induction medium containing either BA alone or in combination with IAA. Therefore, the multiple shoot clumps were transferred to the MS medium devoid of PGRs for shoot elongation (Figure 1(d)).

### 3.2. In Vitro Rooting and Acclimatization

For in vitro root induction, individual shoots were excised from the clusters of elongated shoots and cultured on either half or full strength MS medium supplemented with different auxins such as IAA, IBA, and NAA at 0.5 or 1.0 mg·L^−1^. Within two weeks of culture, roots started to emerge from the shoot base. Among the various root induction media used, IAA and IBA supplemented with the full strength MS medium induced rooting in all shoots (Table 3). Though optimal root induction was observed in the full strength MS medium, the mean number of roots and root length were significantly affected. The half strength MS medium with 0.5 mg·L^−1^ IAA produced maximum number of roots (16.5) with the greatest root length (13.2 cm) (Figure 1(e)). IAA has been considered as the major auxin in plants utilized for adventitious rooting [27]. Though the addition of IBA supported rooting the number of roots produced and the root length were comparatively lesser than IAA treatments. However, supplementation of NAA with both half and full strength media displayed root formation at the nodal region of the stem with basal callusing. In accordance with Arikat et al. [28], NAA exhibited the least rooting potential. Moreover, the auxin NAA might induce stressful environment leading to the formation of nodular roots. Healthy, uniform, and well-rooted plantlets were acclimatized in the greenhouse and produced true leaves after 12 days. Overall, 95% of survival ratio was achieved after five weeks of acclimatization in the greenhouse (Figure 1(f)).

### 3.3. Estimation of Phytochemicals

#### 3.3.1. Total Phenol and Flavonoid Estimation

**Figure 2:**
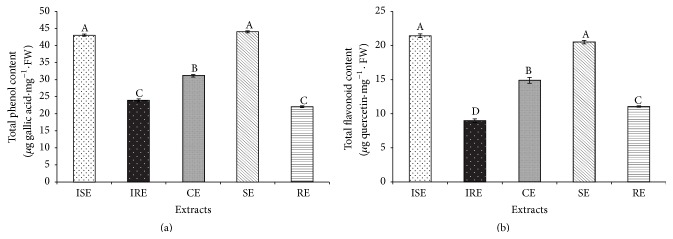
Phytochemical contents of in vitro and in vivo extracts. (a-b) Total phenol and total flavonoid contents present in vitro shoot extract (ISE), in vitro root extract (IRE), callus extracts (CEs), in vivo shoot extract (SE), and in vivo root extract (RE) of* S. kakudensis.* Different letters in one measurement indicate statistically significant difference at *P* ≤ 0.05 by Duncan multiple range test.

The characterization of phytochemical contents of different tissues of* S. kakudensis* is crucial to determine its pharmaceutical importance. The total phenol and flavonoid contents of in vitro shoot extract (ISE), in vitro root extract (IRE), callus extract (CE), in vivo shoot extract (SE), and in vivo root extract (RE) were shown in Figure 2. Among the tissues, shoot contained larger amount of total phenol (Figure 2(a)) and flavonoid contents (Figure 2(b)) than root extracts. According to Amoo et al. [29] the aerial parts of plants are often suitable for the synthesis and accumulation of secondary metabolites in abundance compared to the underground parts. Moreover, the results indicated the accumulation levels of total phenols and flavonoids were nonsignificantly different in ISE and SE. Furthermore, IRE and RE displayed nonsignificant increase in phenol content and RE consisted of higher flavonoids than IRE. Moreover, the CE was also noted with the significant amount of total phenol (31.12 *μ*g GAE·mg^−1^ FW) and flavonoid phytochemical contents (14.88 *μ*g quercetin·mg^−1^ FW). The variation in the production of bioactive compounds in different tissues can be due to the differences in the tissue dependent synthesis and accumulation of phytochemicals, physiological condition of the plant part, and endogenous hormone levels [30]. This result denoted that the contents of phytochemicals in seed derived and in vitro derived plants were similar and the in vitro plantlets can be considered as the alternate for the seed grown plants in terms of therapeutic values.

#### 3.3.2. Assessment of Acacetin Content in Tissue Extracts

**Figure 3:**
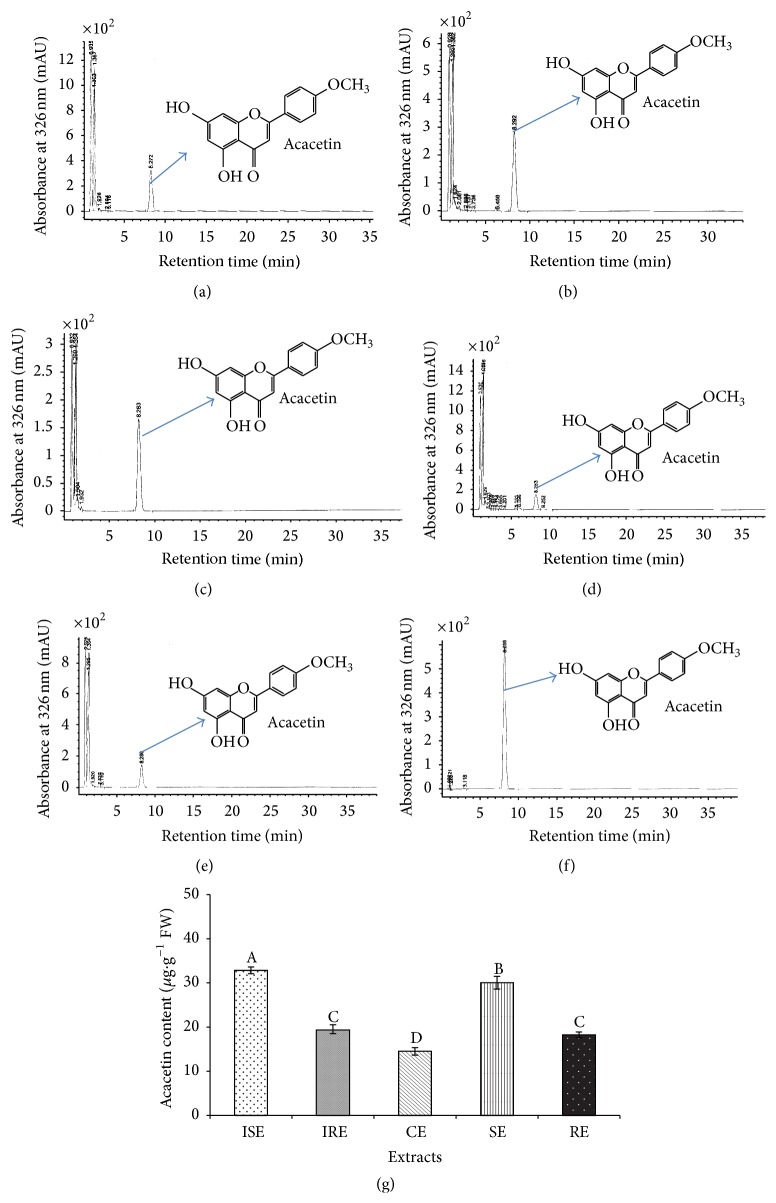
HPLC results of acacetin content estimation in in vitro and in vivo plant extracts. The representative peaks obtained for acacetin in (a) in vitro shoot extract, (b) in vivo shoot extract, (c) callus extracts, (d) in vitro root extract, and (e) in vivo root extract of* S. kakudensis* and (f) reference. (g) The quantification of acacetin in tissue extracts of* S. kakudensis*. Different letters in one measurement indicate statistically significant difference at *P* ≤ 0.05 by Duncan multiple range test.

The highest level of acacetin was estimated in shoot extracts of* S. kakudensis* (Figure 3). Among the extracts, ISE (32.83 *μ*g·g^−1^ FW) and SE (30.05 *μ*g·g^−1^ FW) were noted with the maximum acacetin content followed by the root extracts of both in vitro (IRE: 19.52 *μ*g·g^−1^ FW) and ex vitro (RE: 18.23 *μ*g·g^−1^ FW). The least amount of acacetin was estimated in the callus extract (14.91 *μ*g·g^−1^ FW). Similarly the synthesis of acacetin in leaf tissue was recorded in* Clerodendrum inerme*,* Dioscoreophyllum cumminsii*, and* Ocimum basilicum* [31–33]. The slight increase in the accumulation of acacetin (an individual flavonoid) in in vitro plantlets might be triggered by the plant tissue culture environment and endogenous hormone modulations. According to Shohael et al. [34], the in vitro conditions enhance the secondary metabolite production through the modulation of plant primary metabolism. In addition, synthesis and gene expression levels of the secondary metabolites can be greatly influenced by the nutrient contents and plant hormones employed during the in vitro culture [35]. Likewise, the secondary metabolites production in in vitro plantlets was higher in* Swertia chirayita* than in vivo plantlets [36].

### 3.4. Free Radical Scavenging Potential

#### 3.4.1. Nitric Oxide (NO) Radical Scavenging Assay

**Figure 4:**
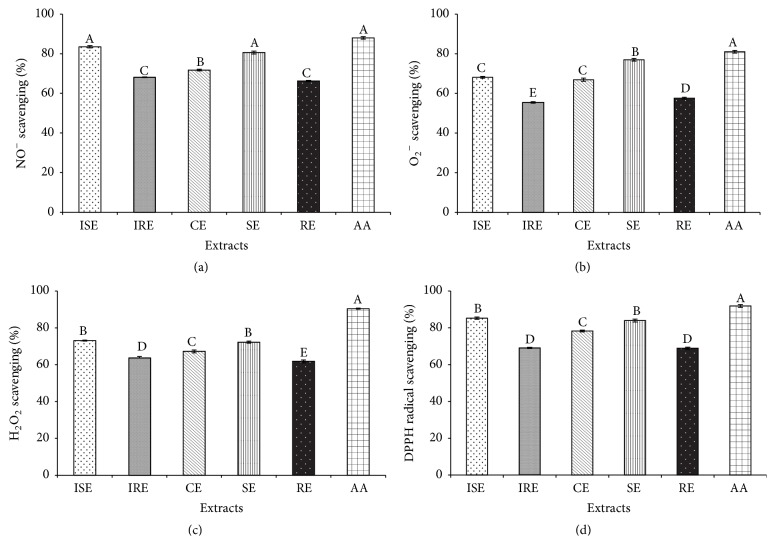
Free radical scavenging potentials of in vitro and in vivo extracts. (a–d) Nitric oxide scavenging, superoxide scavenging, hydrogen peroxide scavenging, and DPPH radical scavenging activities of in vitro shoot extract (ISE), in vitro root extract (IRE), callus extracts (CEs), in vivo shoot extract (SE), and in vivo root extract (RE) of* S. kakudensis* and ascorbic acid (AA). Different letters in one measurement indicate statistically significant difference at *P* ≤ 0.05 by Duncan multiple range test.

The results of NO scavenging assay revealed the substantial ability of the extracts to scavenge NO radical (Figure 4(a)). ISE scavenged 83.49% of NO radicals followed by SE (82.60%). Moreover, the CE exhibited 71.74% of NO inhibition followed by root extracts, respectively. In biological systems, the excess generation of NO radicals leads to various ailments such as chronic inflammatory disorders, carcinogenesis, and atherosclerosis [37]. Therefore, it is highly important to determine the ability of the extracts to inhibit the NO radicals to prevent dreadful diseases.

#### 3.4.2. Superoxide (O_2_^−^) Radical Scavenging Assay

All the tissue extracts inhibited the formation of formazan by scavenging the O_2_^−^ radicals generated by NBT-riboflavin-light complex and the scavenging capacity of the extracts was compared with the positive control, ascorbic acid (Figure 4(b)). Even though all the extracts efficiently inhibited O_2_^−^, SE (76.96%) exhibited excellent O_2_^−^ scavenging. Moreover, no significant difference was observed in the O_2_^−^ scavenging ability of ISE (68.10%) and CE (67.66%). However, the least scavenging potential was noted on the root extracts (RE: 57.62%; IRE: 55.25%). The scavenging ability of ascorbic acid was observed as 81.02%. Superoxide radicals are highly harmful to the cell organelles and superoxide radicals acts as the precursor for the formation of ROS. In addition the larger amount of O_2_^−^ in the cells enhances the dismutation reaction that results in H_2_O_2_ production further leading to the oxidative stress [17]. Therefore, efficient scavenging of O_2_^−^ is inevitable for the antioxidants.

#### 3.4.3. Hydrogen Peroxide (H_2_O_2_) Radical Scavenging Assay

The ability of the tissue extracts to inhibit the hydrogen peroxide radicals was determined and compared with ascorbic acid (Figure 4(c)). The H_2_O_2_ scavenging percentage of the extracts ranged from 61.8 to 73.7%. As expected the ascorbic acid scavenged the greatest amount of H_2_O_2_ radicals (90.42%) followed by ISE (73.07%), SE (72.2%), and CE (67.21%). Moreover, the root extracts displayed H_2_O_2_ elimination activity with moderate scavenging percentage (IRE: 63.6% and RE: 61.8%). In general, H_2_O_2_ is nonreactive but it leads to the production of other toxic free radicals like hydroxyl radicals and causes cell death. Hence, it is necessary for the antioxidant system to scavenge H_2_O_2_ radicals and detoxify the cellular environment. Furthermore, reports suggest that the phenolic compounds readily act as H_2_O_2_ scavengers [17]. Thus, the larger amount of phenolic compounds in the shoot extracts could have attributed to the elevated H_2_O_2_ scavenging capacity.

#### 3.4.4. 2,2-Diphenyl-1-picrylhydrazyl (DPPH) Radical Scavenging Assay

The antioxidant property of the extracts was further confirmed by the DPPH radical scavenging assay. This assay has been widely used in in vitro studies to assess the antioxidant property of the plant extracts. All the extracts displayed DPPH radical scavenging activity (Figure 4(d)). The highest DPPH scavenging has been noted in the ISE (85.25%) and SE (83.93%). Furthermore, the DPPH radical scavenging percentage of CE is recorded as 78.27%. In accordance with other radical scavenging assays IRE (69.41%) and RE (68.90%) exhibited moderate radical inhibition. DPPH is a purple colored stable organic radical which upon accepting an electron from an antioxidant becomes yellow that can be spectrometrically read. Hence, the amount of antioxidants in the reaction mixture can be evaluated by the degree of discoloration [30].

Furthermore, the Pearson correlation analysis revealed the existence of strong positive correlation between the total phenol and flavonoid contents and antioxidant potentials of the tissue extracts (Table 4).

#### 3.4.5. Hydroxyl Radicals Induced Plasmid DNA Strand Scission

**Figure 5:**
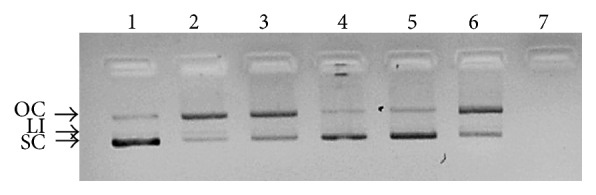
DNA protection potential of in vitro tissues and commercial extracts. Lane 1: positive control (2 *μ*g ascorbic acid + 50 ng DNA). Lane 2: 20 mM H_2_O_2_ + 50 ng DNA + 2 *μ*g in vivo shoot extract (SE). Lane 3: 20 mM H_2_O_2_ + 50 ng DNA + 2 *μ*g in vivo root extract (RE). Lane 4: 20 mM H_2_O_2_ + 50 ng DNA + 2 *μ*g in vitro shoot extract (ISE). Lane 5: 20 mM H_2_O_2_ + 50 ng DNA + 2 *μ*g in vitro root extract (IRE). Lane 6: 20 mM H_2_O_2_ + 50 ng DNA + 2 *μ*g callus extracts (CEs). Lane 7: negative control (20 mM H_2_O_2_ + 50 ng DNA) of* S. kakudensis*. Oc: open circular form, Li: linear form, and Sc: supercoiled form of plasmid DNA.

The DNA strand scission assay illustrated the ability of all the extracts to render protection against the hydroxyl radicals induced DNA damage (Figure 5). During the strand nicking the plasmid DNA will be separated into open circular, linear, and supercoiled form. Based on the intensity of native supercoiled bands the DNA protection capacity was as evaluated and compared with the positive control ascorbic acid (lane 1). The supplementation of SE, RE ISE, IRE, and CE has protected the DNA from the H_2_O_2_ radicals (lanes 2–6). However the in vitro extracts (lanes 4–6) exhibited slightly increased DNA prevention activity compared to the in vivo extracts (lanes 2 and 3). In lane 7 the H_2_O_2_ without any extracts (negative control) induced complete breakage of DNA strands. The excess production of ROS oxidizes the nitrogen bases of the DNA molecules leading to the oxidative damage [22]. Therefore, it is necessary for an antioxidant to protect DNA from the ROS. Thus the extracts were proved to contain strong antioxidant capacity. Overall, the results demonstrated the strong antioxidant properties were attributed in the aerial part of* S. kakudensis* in comparison with the underground part.

## 4. Conclusions

In conclusion, the current endeavor has accomplished a widespread investigation of in vitro propagation by adventitious shoot proliferation, phytochemical evaluation, and free radical scavenging analysis in pharmaceutically important* S. kakudensis*. Therefore, the present work can contribute to the large-scale production of* S. kakudensis* for germplasm conservation and commercial cultivation. In addition, the determination of acacetin content and free radical scavenging potential of* S. kakudensis* tissue extracts can be utilized for the therapeutic benefits. Thus, the difficulty of acquiring the in vivo plants from seeds can be addressed using plant tissue culture approach established in the present study without compromising the medicinal value using in vitro propagated* S. kakudensis* plants.

## Figures and Tables

**Table 1:** Effect of different concentrations of cytokinins on adventitious shoot induction from the nodal explant of *S. kakudensis* Franch.

Treatment	BA (mg·L^−1^)	Kn (mg·L^−1^)	TDZ (mg·L^−1^)	Shoot induction (%)	Number of shoots/explant
1	0.5	—	—	80.5 ± 0.91^d*∗*^	3.1 ± 0.27^f^
2	1.0	—	—	100.0 ± 0.00^a^	6.4 ± 0.14^c^
3	2.0	—	—	100.0 ± 0.00^a^	12.4 ± 0.14^a^
4	3.0	—	—	76.2 ± 0.65^e^	6.2 ± 0.13^dc^
5	—	0.5	—	77.3 ± 0.54^e^	3.0 ± 0.14^f^
6	—	1.0	—	91.4 ± 0.41^b^	5.8 ± 0.20^d^
7	—	2.0	—	100.0 ± 0.00^a^	8.5 ± 0.19^b^
8	—	3.0	—	72.9 ± 0.65^f^	4.2 ± 0.17^e^
9	—	—	0.5	87.1 ± 0.51^c^	6.7 ± 0.17^c^
10	—	—	1.0	76.6 ± 0.71^e^	4.3 ± 0.18^e^
11	—	—	2.0	57.5 ± 0.81^g^	2.5 ± 0.19^g^
12	—	—	3.0	27.2 ± 2.31^h^	1.4 ± 0.14^h^

Values represent the mean ± SE of three replications, each with five explants.

^*∗*^Means followed by same letter(s) within a column are not significantly different (*P* < 0.05).

**Table 2:** Effect of BA and IAA combinations on adventitious shoot induction.

BA (mg·L^−1^)	IAA (mg·L^−1^)	Shoot induction (%)	Number of shoots/explant
0.5	0.5	93.8 ± 0.29^c*∗*^	4.5 ± 0.28^f^
1.0	0.5	100 ± 0.00^a^	10.0 ± 0.47^c^
2.0	0.5	100 ± 0.00^a^	17.4 ± 0.33^a^
3.0	0.5	77.1 ± 1.03^e^	8.2 ± 0.42^d^
0.5	1.0	95.3 ± 0.14^b^	3.6 ± 0.14^f^
1.0	1.0	100 ± 0.00^a^	8.7 ± 0.13^d^
2.0	1.0	100 ± 0.00^a^	14.5 ± 0.51^b^
3.0	1.0	78.6 ± 0.84^d^	6.8 ± 0.47^e^

Values represent the mean ± SE of three replications, each with five explants.

^*∗*^Means followed by same letter(s) within a column are not significantly different (*P* < 0.05).

**Table 3:** Effect of various auxins and strength of the MS medium on in vitro root induction.

Medium	PGR	Conc. (mg·L^−1^)	Rooting (%)	Root length (mm)	Number of roots per explant	Nodular rooting (%)
1/2 strength MS	IAA	0.5	100.0 ± 0.00^a*∗*^	13.2 ± 0.53^a^	16.5 ± 0.33^a^	00.0 ± 0.00^e^
		1.0	99.2 ± 0.27^ba^	11.3 ± 0.22^c^	10.7 ± 0.35^c^	00.0 ± 0.00^e^
	IBA	0.5	96.6 ± 0.35^b^	8.5 ± 0.28^d^	6.6 ± 0.30^f^	00.0 ± 0.00^e^
		1.0	93.9 ± 0.59^c^	10.8 ± 0.27^c^	4.5 ± 0.19^g^	00.0 ± 0.00^e^
	NAA	0.5	86.2 ± 0.52^d^	7.0 ± 0.28^e^	3.9 ± 0.19^hg^	29.5 ± 1.5^d^
		1.0	58.8 ± 2.5^e^	2.9 ± 0.28^g^	3.3 ± 0.22^hi^	79.1 ± 0.56^b^

Full strength MS	IAA	0.5	100.0 ± 0.00^a^	12.3 ± 0.39^b^	9.2 ± 0.27^d^	00.0 ± 0.00^e^
		1.0	100.0 ± 0.00^a^	11.3 ± 0.22^c^	12.7 ± 0.39^b^	00.0 ± 0.00^e^
	IBA	0.5	100.0 ± 0.00^a^	8.5 ± 0.28^d^	6.3 ± 0.22^f^	00.0 ± 0.00^e^
		1.0	100.0 ± 0.00^a^	10.8 ± 0.27^c^	8.4 ± 0.14^e^	00.0 ± 0.00^e^
	NAA	0.5	98.8 ± 0.27^ba^	5.0 ± 0.25^f^	3.9 ± 0.19^hg^	53.6 ± 0.19^c^
		1.0	85.4 ± 1.90^d^	2.9 ± 0.28^g^	2.8 ± 0.32^i^	94.5 ± 0.76^a^

Values represent the mean ± SE of three replications, each with five explants.

^*∗*^Means followed by same letter(s) within a column are not significantly different (*P* ≤ 0.05).

**Table 4:** Correlation coefficient (*r* ^2^) of phytochemicals and antioxidant activities of in vitro and in vivo tissue extracts of *S. kakudensis* Franch.

Extract	Phytochemicals	Correlation coefficient of DPPH	Correlation coefficient of O_2_^−^	Correlation coefficient of H_2_O_2_	Correlation coefficient of NO
ISE	TP	0.95^*∗∗*^	0.96^*∗∗*^	0.99^*∗∗*^	0.90^*∗*^
	TF	0.83^*∗*^	0.95^*∗∗*^	0.96^*∗∗*^	0.74

IRE	TP	0.92^*∗*^	0.92^*∗*^	0.70	0.72
	TF	0.94^*∗*^	0.95^*∗∗*^	0.75	0.77

CE	TP	0.76	0.73	0.98^*∗∗*^	0.72
	TF	0.93^*∗*^	0.90^*∗*^	0.99^*∗∗*^	0.90^*∗*^

SE	TP	0.99^*∗∗*^	0.99^*∗∗*^	0.99^*∗∗*^	0.83^*∗*^
	TF	0.99^*∗∗*^	0.99^*∗∗*^	0.98^*∗∗*^	0.82^*∗*^

RE	TP	0.99^*∗∗*^	0.92^*∗*^	0.95^*∗∗*^	0.82^*∗*^
	TF	0.82^*∗*^	0.95^*∗∗*^	0.92^*∗*^	0.99^*∗∗*^

TP: total phenol content; TF: total flavonoid content; DPPH: 2,2-diphenyl-1-picrylhydrazyl scavenging %; O_2_^−^: superoxide scavenging %; H_2_O_2_: hydrogen peroxide scavenging %; NO: nitric oxide scavenging %.

^*∗*,*∗∗*^Correlation significant at *P* ≤ 0.05 and 0.01, respectively.
